# Blocking Ubiquitin‐Specific Protease 7 Induces Ferroptosis in Gastric Cancer via Targeting Stearoyl‐CoA Desaturase

**DOI:** 10.1002/advs.202307899

**Published:** 2024-03-09

**Authors:** Xiaoqing Guan, Yichao Wang, Wenkai Yu, Yong Wei, Yang Lu, Enyu Dai, Xiaowu Dong, Bing Zhao, Can Hu, Li Yuan, Xin Luan, Kai Miao, Bonan Chen, Xiang‐Dong Cheng, Weidong Zhang, Jiang‐Jiang Qin

**Affiliations:** ^1^ Zhejiang Cancer Hospital Hangzhou Institute of Medicine (HIM) Chinese Academy of Sciences Hangzhou Zhejiang 310022 China; ^2^ Key Laboratory of Prevention Diagnosis and Therapy of Upper Gastrointestinal Cancer of Zhejiang Province Hangzhou Zhejiang 310022 China; ^3^ College of Pharmaceutical Sciences Zhejiang University of Technology Hangzhou Zhejiang 310014 China; ^4^ School of Pharmacy Zhejiang Chinese Medical University Hangzhou Zhejiang 310053 China; ^5^ Hangzhou Institute of Innovative Medicine Institute of Drug Discovery and Design College of Pharmaceutical Sciences Zhejiang University Hangzhou Zhejiang 310058 China; ^6^ Department of Genomic Medicine The University of Texas MD Anderson Cancer Center Houston Texas 77030 USA; ^7^ Institute of Interdisciplinary Integrative Medicine Research Shanghai University of Traditional Chinese Medicine Shanghai 201203 China; ^8^ MOE Frontier Science Centre for Precision Oncology University of Macau Macau SAR 999078 China; ^9^ Department of Anatomical and Cellular Pathology Prince of Wales Hospital The Chinese University of Hong Kong Hong Kong 999077 China; ^10^ School of Pharmacy Naval Medical University Shanghai 200433 China; ^11^ State Key Laboratory for Quality Ensurance and Sustainable Use of Dao‐di Herbs Institute of Medicinal Plant Development Chinese Academy of Medical Science and Peking Union Medical College Beijing 100193 China

**Keywords:** ferroptosis, gastric cancer, SCD, ubiquitination, USP7, USP7 inhibitor

## Abstract

Gastric cancer (GC) presents a formidable global health challenge, and conventional therapies face efficacy limitations. Ubiquitin‐specific protease 7 (USP7) plays pivotal roles in GC development, immune response, and chemo‐resistance, making it a promising target. Various USP7 inhibitors have shown selectivity and efficacy in preclinical studies. However, the mechanistic role of USP7 has not been fully elucidated, and currently, no USP7 inhibitors have been approved for clinical use. In this study, DHPO is identified as a potent USP7 inhibitor for GC treatment through in silico screening. DHPO demonstrates significant anti‐tumor activity in vitro, inhibiting cell viability and clonogenic ability, and preventing tumor migration and invasion. In vivo studies using orthotopic gastric tumor mouse models validate DHPO's efficacy in suppressing tumor growth and metastasis without significant toxicity. Mechanistically, DHPO inhibition triggers ferroptosis, evidenced by mitochondrial alterations, lipid Reactive Oxygen Species (ROS), Malondialdehyde (MDA) accumulation, and iron overload. Further investigations unveil USP7's regulation of Stearoyl‐CoA Desaturase (SCD) through deubiquitination, linking USP7 inhibition to SCD degradation and ferroptosis induction. Overall, this study identifies USP7 as a key player in ferroptosis of GC, elucidates DHPO's inhibitory mechanisms, and highlights its potential for GC treatment by inducing ferroptosis through SCD regulation.

## Graphical abstract

A novel Ubiquitin‐Specific Protease 7 (USP7) inhibitor, DHPO, is identified through in silico screening for gastric cancer (GC) treatment. DHPO demonstrates robust anti‐tumor effects, inducing ferroptosis and effectively suppressing GC growth and metastasis both in vitro and in vivo. Mechanistically, USP7 triggers ferroptosis by targeting Stearoyl‐CoA Desaturase (SCD).

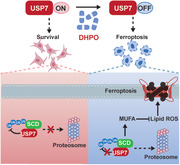

## 1 Introduction

Gastric cancer (GC), also known as stomach cancer, represents a significant global health challenge, ranking as the fifth most prevalent cancer and the third leading cause of cancer‐related mortality. Unfortunately, the prognosis for GC patients is generally poor, with a five‐year survival rate below 30%.^[^ ^1^ ^]^ Traditional chemotherapy, the standard treatment, has limited efficacy and significant side effects.^[^ ^2^ ^]^ Despite advancements in therapies like targeted therapy and immunotherapy, GC remains a formidable challenge to treat. Targeted therapy often faces drug resistance and limited effectiveness, while immunotherapy may not be effective for all GC patients.^[^ ^3^ ^]^ Consequently, novel and effective therapeutic strategies are urgently needed to improve patient outcomes.

Deubiquitinating enzymes (DUBs) play a crucial role in reversing ubiquitination, a process that targets proteins for degradation by the proteasomes or lysosomes. Dysregulation of DUBs has been implicated in various diseases, including cancer,^[^ ^4^ ^]^ neurodegenerative disorders,^[^ ^5^ ^]^ and inflammatory conditions.^[^ ^6^, ^7^ ^]^ Deubiquitinating enzymes can be classified into five subclasses: Ubiquitin‐specific proteases (USPs), Ubiquitin C‐terminal hydrolases (UCH), Ovarian tumor proteases (OTU), Machado‐Joseph disease proteases (MJDs), and JAB1/MPN/Mov34 metalloenzymes (JAMMs). Within the diverse landscape of deubiquitinases, ubiquitin‐specific protease 7 (USP7), also known as herpes virus‐associated ubiquitin‐specific protease (HAUSP), belongs to the largest USP family of DUBs. As for GC, USP7 is interconnected with the development, progression, immune response, and chemo‐resistance of this disease. A recent study revealed that a circular RNA derived from ribosomal protein S19 (circRPS19) upregulates hexokinase 2 (HK2) through USP7‐mediated deubiquitination, consequently inducing aerobic glycolysis in GC cells and fostering GC progression.^[^ ^8^ ^]^ Additionally, USP7 acts upstream to regulate Programmed Death‐Ligand 1 (PD‐L1), a protein involved in cancer immune resistance and growth. Targeting USP7 may weaken the interaction between PD‐1 and PD‐L1, sensitizing GC cells to T‐cell‐mediated killing and enhancing anti‐tumor immunity.^[^ ^9^ ^]^ In Cancer‐associated fibroblasts (CAFs) within GC, USP7 deubiquitinates and stabilizes heterogeneous nuclear ribonucleoprotein A1 (hnRNPA1), facilitating the packaging of miR‐522 into exosomes. This process leads to ALOX15 suppression and decreased lipid‐ROS accumulation in GC cells, and ultimately results in decreased chemo‐sensitivity.^[^ ^10^ ^]^ However, a comprehensive understanding of the mechanisms by which USP7 regulates GC, including novel substrates and signaling networks, remains elusive.

Targeting USP7 has emerged as a promising therapeutic strategy in different types of cancers, including GC.^[^ ^11^ ^]^ Recent efforts have focused on identifying USP7 inhibitors. Categorizing these inhibitors based on their binding sites, USP7 inhibitors can be classified into three types: catalytic site covalent inhibitors, allosteric site covalent inhibitors, and allosteric site reversible inhibitors. The initial discovery of the catalytic site covalent inhibitors dates back to 2011 when Mikael Altun and colleagues successfully developed a selective small‐molecule inhibitor P05091 for USP7 using dynamic chemical proteomics. Subsequent medicinal chemistry optimization led to the creation of P22077. Examples of catalytic site covalent inhibitors also include P50429,^[^ ^12^ ^]^ HBX19818 and HBX28258,^[^ ^13^ ^]^ and GNE‐3086 and GNE‐3093.^[^ ^14^ ^]^ These inhibitors irreversibly inhibit USP7 enzymatic activity by nucleophilic attack on Cys223. C9, a quinazolin‐4‐one derivative synthesized by Li and collaborators, exhibited a low micromolar potency for inhibiting USP7 catalytic activity, thus decreasing MDM2 protein level and stabilizing p53 in cancer cells. Molecular docking studies predicted that C9 would form hydrogen bond interactions with the Met407 of USP7.^[^ ^15^ ^]^ As of now, allosteric site covalent inhibitors of USP7 can be broadly categorized into two classes, represented by GNE‐6776 and FT‐671. GNE‐6776, developed by Kategaya et al. in 2017 using nuclear magnetic resonance‐based screening and structure‐based design, exhibits moderate activity and selectivity, resulting in a relatively limited and slow development of this class of allosteric site inhibitors.^[^ ^16^ ^]^ In the same year, Turnbull and colleagues introduced a novel class of allosteric inhibitors, including FT827 as a covalent inhibitor, and FT671 as a non‐covalent inhibitor. Both inhibitors display exceptional selectivity for USP7.^[^ ^17^ ^]^ Additionally, Zeng et al. reported allosteric inhibition of USP7 by EB, a small molecule that targets the noncatalytic HUBL domain.^[^ ^18^ ^]^ Although USP7 inhibitors with various scaffolds are continuously being discovered or designed, as of now, none of them has entered the clinical stage.

In this study, our objective is to develop novel and specific USP7 inhibitors for the treatment of GC. Using identified small molecule inhibitors as experimental probes, we aim to explore new mechanisms and substrates associated with the occurrence and development of GC involving USP7. Furthermore, we seek to elucidate the underlying molecular mechanisms of the newly identified USP7 inhibitor and assess its potential implications in the treatment of GC.

## 2 Experimental Section

### 2.1 Cell Lines, Chemicals, and Clinical Samples

Human GC cell lines, including MGC803, MKN1, HGC‐27, AGS, NUGC4, and AZ521 were purchased from the American Type Culture Collection (ATCC). All cell lines were cultured at 37 °C with 5% CO_2_ in RPMI‐1640 medium (GIBCO) supplemented with 10% fetal bovine serum (FBS, GIBCO, Thermo Scientific), penicillin (100 IU/ml), and streptomycin (100 mg/mL). The USP7 inhibitor FT671 and P5091 were purchased from TargetMol (FT671, cat#1959551‐26‐8; P5091, cat# 882257‐11‐6). The investigated compounds, including DHPO, were obtained from a natural product library established in Prof. Wei‐Dong Zhang's laboratory, with purity being >95%.^[^ ^19^, ^20^ ^]^

A total of 128 pairs of fresh‐frozen GC tumor tissues and para‐tumor samples were collected from patients who underwent GC surgery in the Department of Gastric Surgery at Zhejiang Cancer Hospital (Hangzhou, China). Informed consent forms were signed in advance. Follow‐up was conducted in time to record the survival time of patients. All studies associated with clinical samples and data were carried out following the principles of the Declaration of Helsinki and were approved by the Ethics Committee of Zhejiang Cancer Hospital (study number: IRB‐2021‐456).

### 2.2 Molecular Docking

The crystal structure of USP7 was obtained from the RCSB Protein Data Bank (PDB ID: 5UQV) and preprocessed in Schrödinger's (2021‐3) Protein Preparation Wizard, including the removal of water, alternate position of residues, addition of hydrogens, assignment of bond orders, optimization of H‐bonds, and restrained minimization of energy. DHPO was prepared using the Ligprep module to generate the possible ionization states and three‐dimensional conformations. Lastly, the compound was docked to the pocket of USP7 using the Covalent Docking module. C300 was selected as a covalently bound amino acid, the length of the pocket in each direction of XYZ was set to = 20 Å, and other preparation parameters were set to default. Finally, the optimal ligand pose was selected based on the docking score.

### 2.3 MicroScale Thermophoresis (MST)

DHPO's binding affinity to purified wild‐type USP7 was evaluated using Nanotemper Technologies' Monolith NT.115 system. Protein and Red‐Tris‐NTA dye affinity tests were conducted to determine the affinity and labeling efficiency of the His‐labeling dye for the His‐tagged protein. A 50 nm RED‐tris‐NTA 2nd Generation dye solution was prepared by mixing dye (5 µm) with PBS‐T. The dye solution was then mixed with unlabeled protein at different dilutions and further incubated to allow binding. Samples were loaded into capillaries and measurements were taken using appropriate power settings. MO.Affinity Analysis software was used for data analysis. Results showed a good affinity (>10 nm) between the USP7 protein and the dye. Further protein and small molecule affinity testing was performed. For protein labeling, purified recombinant proteins were dialyzed into 1× PBS and adjusted to 200 nm concentration. Protein labeling was performed according to Nanotemper's protocol using the Protein Labeling Kit RED‐tris‐NTA. Stock compounds (10 mm) were prepared for the MST assay. Labeled proteins were mixed with unlabeled compounds at different concentrations and loaded into premium capillaries. Measurements were taken using specific power settings and Nanotemper analysis software was used to determine the *K*_d_ value.

### 2.4 Drug Conjugate Site Analysis

For intact protein analysis, recombinant USP7 alone as well as USP7 mixed with DHPO were diluted to 1 mg/mL in 0.1% formic acid (FA). A total of 2 µg of protein was injected for each LC/MS run. An Agilent 1290 Infinity II LC system coupled with a 6545 QTOF mass spectrometer (Agilent, Santa Clara, CA, USA) was used. Intact protein samples were separated using an Agilent PLRP‐S column (1.0 × 50 mm, 5 µm) with a 12 min gradient (holding at 5% B for 5 min, increasing from 5% to 95% B over 5 min, holding at 95% B for 2 min) at a flow rate of 0.300 mL min^−1^. Mobile Phase A consisted of water with 0.1% formic acid, while Mobile Phase B consisted of acetonitrile with 0.1% formic acid. The mass spectrometry instrument parameters were set as follows: a dry gas flow rate of 10.0 L min^−1^ at 325 °C, a nebulizer pressure of 50 psig, a capillary voltage of 4.5 kV, and a scan range of 500 to 3200 m/z at 1 Hz. The LC/MS raw data obtained from the intact mass analysis was processed using MassHunter BioConfirm software (Version 10.0, Agilent, Santa Clara, CA, USA) for deconvolution of the intact protein masses.

For drug conjugate site analysis, a 100 µg sample was loaded onto 10k Microcon filtration devices (Millipore) and centrifuged at 14000 ×g for 30 min at 4 °C to remove unbound drug compounds. Subsequently, 200 µL of 50 mm NH_4_HCO_3_ was added, and the sample was centrifuged again at 14000 ×g for 30 min. Finally, 100 µL of 50 mM NH_4_HCO_3_ and Trypsin (enzyme‐to‐protein ratio of 1:25) were added to the sample, which was then incubated at 37 °C for 16 h. A 20 µg portion of the digested peptide mixture was desalted using a C18 tip for further analysis. For the analysis of enzyme digestion of peptide mixtures, an Easy nano‐LC1000 system with a self‐packed column (75 µm × 150 mm; 3 µm ReproSil‐Pur C18 beads, 120 Å, Dr. Maisch GmbH, Ammerbuch, Germany) was used. The peptides were eluted using a gradient (2%–90% mobile phase B) over a 60‐minute period at a flow rate of 300 nL min^−1^. The mobile phase A consisted of 0.1% formic acid in water, while mobile phase B consisted of 0.1% formic acid in acetonitrile. The eluted peptides were analyzed by a nano‐ESI Q Exactive mass spectrometer in data‐dependent mode. Each full MS scan covered a range of m/z 300 to 1600, followed by MS/MS analysis of the 10 most intense ions. The parameters for MS/MS analysis included precursor ion charges of ≥ +2, a 2 Da isolation window for precursor ions, and a normalized collision energy of 27 in HCD. Dynamic Exclusion was set to 30 s. The full mass and subsequent MS/MS analyses were scanned in the Orbitrap analyzer with a resolution of 70000 and 17500, respectively. The LC/MS‐MS raw data obtained from peptide analysis was processed using pFind software (Version 3.1.5) with a false discovery rate (FDR) <0.01 at both peptide and protein levels. Trypsin/P was selected as the digestive enzyme with allowance for two potential missed cleavages. The search included variable modifications of methionine oxidation and N‐terminal acetylation, as well as cysteine drug conjugation (molecular formula, monoisotopic mass).

### 2.5 In vitro Anti‐GC Activity Assay

#### 2.5.1 Cell Viability Assay

Measurements of cell viability at different drug concentrations were performed. MGC803 and MKN1 cell lines were seeded in 96‐well plates (3×10^4^ cells/mL) at 37 °C overnight with 5% CO_2_ and then incubated with various concentrations of DHPO (0, 0.78, 1.56, 3.12, 6.25, 12.5, and 25 µm) at 37 °C for 72 h. Wells containing only the complete medium were used as the blank control group, and wells containing tumor cells suspended in the complete medium were used as a control group. Thiazolyl blue tetrazolium bromide (0.5 mg/mL) was added to each well. After incubation for 4 h, using a Multiskan Ascent microplate photometer, the absorbance was measured at 450 nm wavelength. The control group cells were regarded as having a 100% survival rate. The percentage of growth inhibition was calculated as cell growth inhibition (%) = (treated OD‐blank OD)/(control OD‐blank OD) × 100%. The concentration required for a 50% inhibition of viability (IC_50_) was then determined.

#### 2.5.2 Colony Formation Assay

MGC803 and MKN1 cell lines (500 cells/well) were seeded in 6‐well flat‐bottomed plates and incubated for 24 h. Using different concentrations of DHPO as the experimental group and complete medium as the control group, MGC803 and HGC27 cells were tested. Cells were treated with different concentrations (0, 1, or 2 µm) of DHPO. After 10–14 days, colonies were fixed with 4% paraformaldehyde for 15 min, washed with deionized water, stained with 0.5% crystal violet for 20 min, washed again with deionized water, and photographed.

#### 2.5.3 Wound Healing Assay

Cells were cultured to form a confluent monolayer, followed by the creation of a uniform scratch with a sterile pipette tip. Subsequently, the cells were treated with DHPO at the specified concentrations (0, 1, and 2 µm), and phase‐contrast images of the scratch were captured at 0, 12, and 36 h. Image analysis software was utilized to measure the wound closure area, and the percentage of closure was calculated in comparison to the initial scratch area. Statistical analysis was performed to assess the significance of observed differences between the treated and control groups.

#### 2.5.4 Transwell Invasion Assay

Cells were seeded into the upper Matrigel‐filled chamber of a 24‐well transwell plate with 200 mL of serum‐free medium. The MGC803 and MKN1 cell density was adjusted to 2 × 10^5^ cells/mL. Then, DHPO with different concentrations (0, 1, or 2 µM) was added to the cells. Simultaneously, 500 mL of 10% FBS‐supplemented medium was added to the lower chamber. Cells were removed from the upper Matrigel chamber membrane using a cotton swab after 24 h of incubation with DHPO and the chambers were further fixed with methanol and stained with Giemsa. Three fields per chamber were analyzed by light microscopy for cell invasion quantification. The experiment was repeated three times.

### 2.6 Transmission Electron Microscopy (TEM)

NUGC4 cells were subjected to the indicated treatment and then underwent centrifugation at 5000 rpm for 5 min following trypsinization. Afterward, the cells were fixed with 2.5% glutaraldehyde at 4 °C for a duration of 2 h. Subsequent to fixation, the cells were postfixed with 1% osmium tetroxide under the same temperature conditions for 1 h. The cells were then dehydrated using a series of alcohol and acetone solutions of increasing concentration. Following dehydration, the cells were embedded in SPI‐Pon 812 (Electron Microscopy Sciences, Hatfield, PA, USA). Ultrathin sections were obtained by employing a Leica EM UC7 Ultramicrotome (Leica Microsystems, Buffalo Grove, IL, USA) and stained with uranyl acetate and lead citrate. TEM images were captured utilizing a JEM‐2100Plus Electron Microscope (JEOL Ltd. Tokyo, Japan).

### 2.7 Lipid ROS Measurement

The digested cells were transferred from a 10‐cm dish into a centrifuge tube and three cycles of centrifugation were performed with PBS washing. The cells were resuspended in 1 mL of PBS, and 1 uL of C11‐BODIPY 581/591 probe (Invitrogen, USA) was added to each tube, which was placed in an incubator for 5 min. Following the incubation period, the centrifugation and washing with PBS were repeated three times. The cells were resuspended in 500 µL of PBS, passed through a sieve, and then proceeded on a Beckman Coulter (United States) with a collection of 10000 cells at low speed using the FITC channel.

### 2.8 Malondialdehyde (MDA) Content Assay

A total of 5 × 10^6^ cells were lysed using 500 µL of lysis buffer. The lysates were centrifuged at 10000‐12000 g for 10 min to obtain the supernatant. The protein concentration of the supernatant was determined to facilitate subsequent calculations of intracellular MDA content. MDA levels were assessed using the Lipid Peroxidation MDA Assay Kit (S0131, Beyotime, China), following the manufacturer's protocol. Briefly, 100 µL of the sample was combined with different concentrations of standards to construct a standard curve in a centrifuge tube. Subsequently, 200 µL of the MDA detection working solution was added, and the mixture was thoroughly mixed before heating at 100 °C in a metal bath for 15 min. After cooling to room temperature in a water bath, the samples underwent centrifugation at 1000 g for 10 min. The resulting supernatant (200 µL) was transferred to a 96‐well plate, and the absorbance was measured at 532 nm using a microplate reader.

### 2.9 Measurement of Irons

The iron colorimetric assay kit (E‐BC‐K880‐M, Elabscience, Wuhan) was used to detect total intracellular irons. Briefly, cells were cultured in 6‐well plates with a density of 1 × 10^6^ cells per well. Following incubation with DHPO for 24 h, cells were harvested and lysed. The supernatant was collected by centrifugation to detect the level of irons.

### 2.10 Stable and Transient Transfection

The construction and procurement of lenti‐shUSP7, and their respective control vectors were done by GeneChem (Shanghai, China). Infections were carried out following standard procedures. After 48 h of lentivirus infection, positive cells were selected in the presence of puromycin obtained from Sigma‐Aldrich Corp. (St. Louis, MO, USA). The concentration of puromycin was gradually reduced to a maintenance level, and the screening and amplification process continued. Cells were collected for Western Blot identification. After 2–4 weeks, a stable mixed clone strain was obtained. The siRNA molecules targeting USP7 were synthesized, purified, and purchased from GenePharma (Shanghai, China). Transfection of the siRNA into cells was performed using Lipofectamine 2000 from Invitrogen (Carlsbad, CA, USA), following the manufacturer's instructions. The sequences of the siRNAs used were as follows: siUSP7#1: 5′‐UGUAUCUAUUGACUGCCCUTT‐3′; siUSP7#2: 5′‐CGUGGUGUCAAGGUGUACUTT‐3′.

### 2.11 Western Blotting

Cells were lysed with Radioimmunoprecipitation assay buffer (RIPA buffer) supplemented with protease inhibitor cocktails from Selleck Chemicals, and protein was extracted. The protein concentration was determined using a Bradford assay kit from Thermo Fisher Scientific. Equal amounts of protein were loaded onto SDS‐polyacrylamide gel electrophoresis (SDS‐PAGE), with β‐actin serving as the loading control. The proteins were separated by electrophoresis, transferred to a nitrocellulose membrane, and then blocked with a blocking solution. Primary antibodies specific to the target proteins were incubated with the membrane, followed by washing and incubation with secondary antibodies conjugated to horseradish peroxidase. Protein bands were visualized using chemiluminescent substrates and captured using a chemiluminescence imaging system. The antibodies used included anti‐USP7 antibody (Cell Signaling Technology, cat#4833), anti‐SCD antibodies (Invitrogen, cat#MA5‐27542; abcam, cat#ab39969), anti‐HA antibody (Beyotime, cat#AH158), anti‐β‐actin monoclonal antibody (Immunoway, cat#YM3028), and anti‐rabbit IgG (Cell Signaling Technology, cat#707).

### 2.12 RNA Extraction and Quantitative Real‐Time PCR

The cells were washed with PBS buffer three times and then incubated with 1 mL Trizol lysis buffer for 10 min. The cell lysates were collected and further incubated with 0.2 mL chloroform for 3 min, followed by centrifugation at 12000 g, 4 °C for 15 min. The upper colorless aqueous phase was carefully transferred into a new 1.5 mL EP tube, gently mixed with an equal volume of isopropyl alcohol, and stood at room temperature for 10 min. After centrifugation at 12000 g, 4 °C for 10 min, the supernatant was discarded. The pellet was washed with ice‐cold 75% ethanol and dissolved in an appropriate amount of DEPC (RNase Free) water. The concentrations and purities of all RNA samples were determined, and the reverse transcription was performed to synthesize cDNA using HiScript 1st Strand cDNA Synthesis Kit (Vazyme). Gene expression levels were determined using 2 × Phanta Max Master Mix (Dye Plus) (Vazyme).

### 2.13 Immunoprecipitation (IP)

IP extracts were prepared using RIPA buffer supplemented with protease inhibitor cocktails (Selleck Chemicals, United States). These extracts were then incubated with specific antibodies at 4 °C for 12 h on a rotating platform. Following this, protein A/G‐magnetic beads from MCE were added to the mixtures and incubated at 4 °C for 1 h on a rotator. Upon completion of the incubation, the beads underwent four washes in IP buffer and were subsequently boiled in 1× loading buffer. Protein samples were analyzed using SDS‐PAGE to assess the interactions between the target proteins. Western blotting assays were employed to detect the proteins of interest within the co‐IP products. Furthermore, the ubiquitin assay was carried out under denaturing conditions.

### 2.14 In Vivo Anti‐GC Efficacy Studies

All animal care and experimental procedures in this study were approved by the Institutional Animal Care and Use Committees of Zhejiang Cancer Hospital (approval number: 2022‐03‐038). This research followed the rules of 3R's (reduction, replacement, and refinement).

#### 2.14.1 Orthotopic Tumor Mouse Models

Male 4‐ to 5‐week‐old SCID mice were obtained from the Shanghai SLAC Laboratory Animal Co., Ltd. Two GC cell lines, MGC803‐luc and MKN1‐luc, were used in the study. Briefly, both cell lines were harvested and suspended in PBS at a concentration of 1 × 10^7^ cells/100 µL and subcutaneously injected to establish xenograft mouse models. The formed xenograft tumors were dissected and cut into 1 mm^3^ tissue fragments, and then inserted into the surface of the serosa of the stomach as described previously.^[^ ^21^ ^]^ The orthotopic tumor‐bearing mice were grouped and treated in the following way. For MGC803 tumor‐bearing mice, DHPO was administrated by intraperitoneal injection at a dose of 5 or 10 mg kg^−1^ day^−1^ for 5 weeks. For MKN1 tumor‐bearing mice, DHPO was administrated by intraperitoneal injection at a dose of 10 mg kg^−1^ day^−1^ for 5 weeks. Tumor growth was monitored weekly through bioluminescence imaging to assess drug potency. Additionally, bioluminescence imaging was used to evaluate metastatic potential by tracking cancer cell accumulation in organs such as the liver, peritoneum, and spleen at the end of the study. Mouse body weight was monitored to assess toxicity. At the end of the experiment, vital organs were harvested for Hematoxylin and Eosin staining (HE) to evaluate histological changes.

#### 2.14.2 Patient‐Derived Xenografts (PDX)

PDX animal models were established by Oncocare Co., Ltd (Hangzhou, China). Briefly, 5–6 weeks old BALB/c nude mice were obtained and acclimated for one week in a specific pathogen‐free environment. GC PDX were collected and transplanted into the mice by subcutaneously implanting small tumor pieces (≈2–3 mm^3^) into their flanks. Once tumors reached ≈50 mm^3^, mice were randomly divided into the vehicle group, DHPO treatment groups (5 or 10 mg kg^−1^ day^−1^), and cisplatin treatment group (5 mg kg^−1^ week^−1^). Tumor volume was measured every 3 days using a caliper, and relative tumor volume and growth rate were calculated to assess drug efficacy. Survival time was also monitored as an indicator of anti‐tumor efficacy, and mice were euthanized when showing signs of morbidity or excessive tumor burden. Daily body weight measurements were recorded to assess drug toxicity. Survival curves were generated using the Kaplan‐Meier method, and differences between groups were compared using the log‐rank test. Additionally, tumor tissues were harvested at the end of the study for immunohistochemistry (IHC) staining and Western blot analysis to evaluate the expression of USP7, Stearoyl‐CoA Desaturase (SCD), and 4‐Hydroxynonenal (4‐HNE). Antibodies used for IHC are as follows: anti‐USP7 antibody (Cell Signaling Technology, cat#4833), anti‐SCD antibody (Atlas Antibodies, cat# HPA012107), and anti‐4‐HNE antibody (Bioss, cat#6313R).

### 2.15 Bioinformatic Analysis

#### 2.15.1 Public Data Sources

The gene dependencies of gastric cancer (GC) cell lines were obtained from the DepMap website using CRISPR (DepMap Public 23Q2+Score, Chronos) and RNAi (Achilles+DRIVE+Marcotte, DEMETER2) datasets. These datasets can be downloaded from the DepMap website (https://depmap.org/portal/download/). The RNA‐seq data for GC from The Cancer Genome Atlas (TCGA) dataset were downloaded from the UCSC Xena website (http://xena.ucsc.edu). Specifically, the dataset used was GDC TCGA Stomach Cancer (STAD). The RNA‐seq data for the Asian Cancer Research Group (ACRG) dataset was obtained from the GEO website (https://www.ncbi.nlm.nih.gov/geo/) under accession number GSE66254. The RNA‐seq data for the Zhejiang dataset was obtained from the Sequence Read Archive (SRA) database under the accession number PRJNA788008. The scRNA‐seq data was downloaded from GEO under accession number GSE234129.

#### 2.15.2 RNA‐Seq Analysis

MGC803 cells were plated in 10‐cm dishes and subjected to the specified treatment. Following the treatment, the cells were washed three times with PBS. Then, the cells were lysed using Trizol reagent at room temperature, and the samples were kept on dry ice. An RNA‐seq transcriptome library was prepared using the TruSeq RNA sample preparation Kit from Illumina (San Diego, CA), utilizing 1 mg of total RNA. The RNA‐seq data were generated by LC‐Bio Technology Co., Ltd (Hangzhou, China) and subsequently analyzed using the free online platform provided by Lianchuan Cloud Platform. Differential expression analysis was performed using the DESeq package in the R platform, utilizing the default configuration. Furthermore, gene set enrichment analysis (GSEA) was carried out using hallmark gene sets from MSigDB.

#### 2.15.3 LC‐MS/MS Analysis

LC‐Bio Technology Co., Ltd (Hangzhou, China) generated proteomic data for MGC803 cells treated with DHPO or DMSO. Three replicates were set for each group. The cells were washed three times with pre‐cooled PBS, collected in a centrifuge tube, and centrifuged at 1000 g, 4 °C for 5 min. The supernatant was then removed. Protein extraction, enzyme digestion, liquid chromatography‐mass spectrometry tandem analysis, and bioinformatics analysis techniques were used to conduct quantitative proteome research on the samples. Differential expression analysis was conducted utilizing the t‐test, with pathway enrichment analysis carried out using the Kyoto Encyclopedia of Genes and Genomes (KEGG) gene sets.

### 2.16 Statistical Analysis

Statistical analysis was performed using GraphPad Prism version 8.0 software. Results were presented as mean ± standard error of the mean (SEM). Statistical significance was set at *p* <0.05.

## 3 Results

### 3.1 Identification of USP7 as an Oncogenic Driver in GC

**Figure 1:**
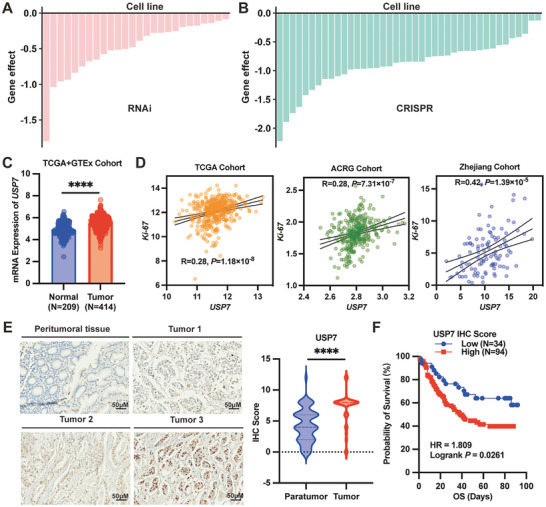
USP7 is an oncogenic driver in gastric cancer (GC). A,B) Barplots showing the USP7 essentiality in GC cell lines from publicly available RNAi screening (A) and CRISPR‐Cas9 screening (B). C) mRNA expression of USP7 in human GC tissues and normal tissues from the TCGA and GTEx cohort. D) Correlation analysis of mRNA expression of USP7 and Ki‐67 in human GC tissues in the TCGA, ACRG, and Zhejiang cohorts. E) The representative IHC images and statistical results of USP7 expression in para‐cancerous and tumor tissues of GC patients. F) Survival analysis of 128 GC patients with high USP7 expression compared to low expression.

We conducted a comprehensive analysis of siRNA and CRISPR data derived from the DepMap datasets, focusing specifically on USP7. Briefly, we extracted the dependency scores of USP7 from these datasets, indicative of the extent to which a gene is crucial for the survival of a cell line. A score of −1 denotes complete genome lethality, while a score of 0 implies no discernible effect. Our analysis unveiled a significant enrichment of USP7 in GC in two datasets (**Figure** **1**A,B; Figure S1A,B, Supporting Information), underscoring its potential relevance to the disease. Subsequently, we explored the alteration in USP7 abundance in human GC tissues using CVCDAP, a publicly accessible cancer informatics database.^[^ ^10^ ^]^ Our analysis revealed a higher mRNA expression of USP7 in GC tissues compared to normal gastric tissues (Figure 1C). To assess the impact of USP7 on GC cell proliferation, we calculated the Pearson correlation coefficient between the mRNA expression of USP7 and Ki‐67 (a marker of cellular proliferation) across all samples in the TCGA, ACRG, and Zhejiang cohorts. Notably, our analysis demonstrated a positive correlation between USP7 and Ki‐67 in all three datasets (Figure 1D; Figure S1C,D, Supporting Information). Collectively, our findings suggest a potential functional linkage between USP7 and the regulation of Ki‐67 expression in GC. Furthermore, we explored the clinical relevance of USP7 in GC using our in‐house cohort of 128 patients. Employing tissue microarray IHC, we observed upregulation of USP7 in GC tissues relative to para‐tumor tissues (Figure 1E). Moreover, the elevated expression of USP7 is correlated with poor prognosis in GC (Figure 1F). These observations underscore the clinical significance of USP7 in GC and highlight its potential as a therapeutic target.

### 3.2 Structure‐Based in silico and GC Cell Screens Identified a Novel USP7 Inhibitor

**Figure 2:**
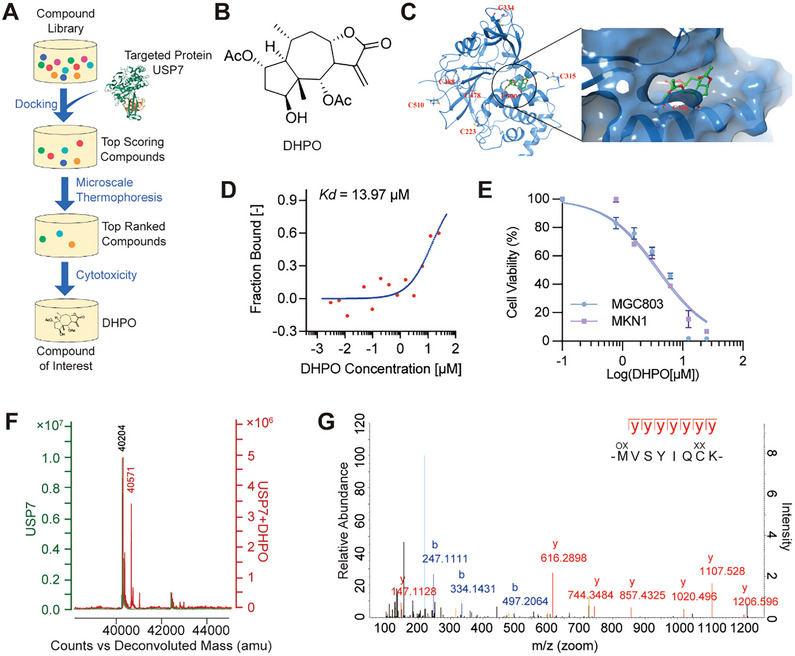
Identification of a novel USP7 inhibitor DHPO. A) Overview of in silico screening of small‐molecule inhibitors of USP7. B) Structure of DHPO. C) Molecular docking of DHPO in the allosteric site of human USP7. D) MicroScale Thermophoresis (MST) assay showing binding affinity of DHPO and purified USP7 protein. E) The anti‐proliferative effects of DHPO on MGC803 and MKN1 were assessed using CCK‐8 assay after 72 h of treatment. F) Linear MS spectra of USP7 without or with DHPO incubation show a mass shift of USP7 with the addition of DHPO. G) MS/MS analysis of DHPO‐bound peptide 294–301.

Given the current lack of U.S. Food and Drug Administration (FDA)‐approved inhibitors for USP7 in clinical use, we endeavored to discover a novel USP7 inhibitor for treating GC. Herein, we performed in silico structure‐based screens followed by cytotoxic GC cell assays (**Figure** **2**A). Our in‐house library of more than 3000 compounds was screened in silico against crystal structures of USP7, which represent the dominant conformations of the active site loop. The compound with a favorable docking score was selected for cytotoxicity in MGC803 cells at concentrations of 0, 1, 10, and 100 µM. Finally, one compound termed DHPO (2α,6α‐diacetoxy‐4β‐hydroxy11^[^ ^13^ ^]^‐pseudoguaien‐12,8α‐olide, molecular weight = 366 g mol^−1^) (Figure 2B) emerged as the top candidate from both screens as binding with USP7 and having cytotoxic effects in GC cells. According to computational modeling, DHPO is predicted to be located within the cleft of the USP7 allosteric site. It is positioned in such a way that its reactive center is close to Cys^300^, which is within a pocket ≈12 Å away from the catalytic triad. The catalytic triad is situated at the interface of the USP7 catalytic domain palm, fingers, and thumb sub‐domains (Figure 2C). This arrangement facilitates the formation of a covalent bond through a Michael addition mechanism. Importantly, this binding interaction sterically hinders the binding of ubiquitin and prevents the transition of the α5 helix in the USP7 catalytic domain to its active conformation.^[^ ^16^, ^22^ ^]^ The direct binding of DHPO and USP7 was confirmed by an independent assay called MST, in that a robust binding curve was detected with a *K*_d_ at 13.97 µm (Figure 2D). The compound was further evaluated for its anti‐GC activity. Our results showed that treatment with DHPO led to a concentration‐dependent decrease in cell viability in MGC803 and MKN1 cells (Figure 2E). At the highest concentration tested (25 µm), cell viability was reduced by over 95% after 72 h of treatment. Consistent with the model of docking, LC/MS analyses showed that DHPO bound to recombinant USP7 protein. MALDI‐TOF‐MS revealed that the mass shift of wild‐type USP7 protein was ≈366 Da when incubated with DHPO resulting from a Michael addition (Figure 2F), which is the predicted molecular weight of the chemical adduct of DHPO. To further identify the residues that are critical for the binding process, we made an impartial MS analysis to detect cysteine residues modified by DHPO in vitro. The MS analysis of chymotryptic peptide covering Cys^300^ (amino acid 294–301) showed an increase of 366 in mass, demonstrating that Cys^300^ was alkylated by DHPO (Figure 2G). Collectively, these results suggest a covalent binding of DHPO to the catalytic Cys^300^ in GC cells.

### 3.3 DHPO Suppressed Growth and Metastasis of GC In Vitro and In Vivo

**Figure 3:**
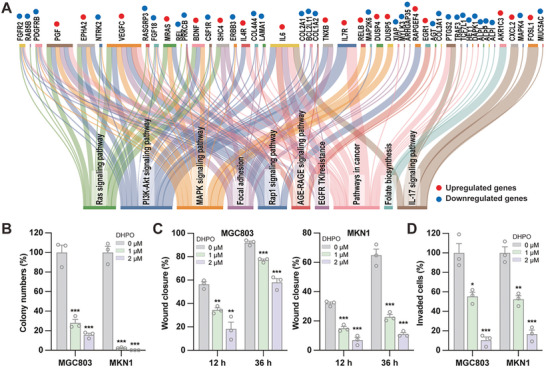
The USP7 inhibitor DHPO exerts anti‐gastric cancer (GC) activity in vitro. A) RNA‐seq and pathway enrichment analysis in GC cells treated with DHPO or DMSO. B) Colony formation experiments were performed to quantify and analyze cell colonies in MGC803 and MKN1 cell lines treated with DHPO at concentrations of 0, 1, and 2 µm. C) Wound healing assays were performed to evaluate cell migration after 12 h and 36 h of DHPO treatment (0, 1, and 2 µM) in MGC803 and MKN1 cell lines, with wound closure percentage calculated. D) Transwell assays were performed to quantify the effects of DHPO (0, 1, and 2 µm) on the invasive ability of MGC803 and MKN1 cell lines.

To further explore the anti‐GC efficacy of DHPO, RNA‐seq analysis was performed on MGC803 cells, and DEGs between treated and untreated cells were identified (*Padj* < 0.05, log2 fold change >1). In total, 172 upregulated and 266 downregulated genes were significantly differentially expressed. Pathway enrichment analysis was conducted to identify the potential signaling pathways regulated by DHPO. The analysis revealed that DEGs were linked to cell proliferation‐related pathways (such as Ras signaling, PI3K‐Akt signaling, MAPK signaling, and AGE‐RAGE signaling), as well as metastasis‐related pathways (including Focal adhesion and Rap1 signaling) (**Figure** **3**A). These findings suggested that DHPO inhibited cell growth and metastasis in GC cells. As illustrated in Figure 3B, DHPO significantly reduced the clonogenic potential of GC cells in the colony formation assay. The number and size of colonies were markedly decreased in cells treated with the inhibitor compared to the control group (Figure 3B). In the wound healing assay, DHPO delayed the closure of the wound compared to the control group (Figure 3C). Similarly, in the transwell assay, the invasive properties of GC cells were significantly reduced in cells treated with the DHPO compared to the control group (Figure 3D). These findings demonstrate that the USP7 inhibitor DHPO has a potent inhibitory effect on the tumorigenic potential of GC cells in vitro.

**Figure 4:**
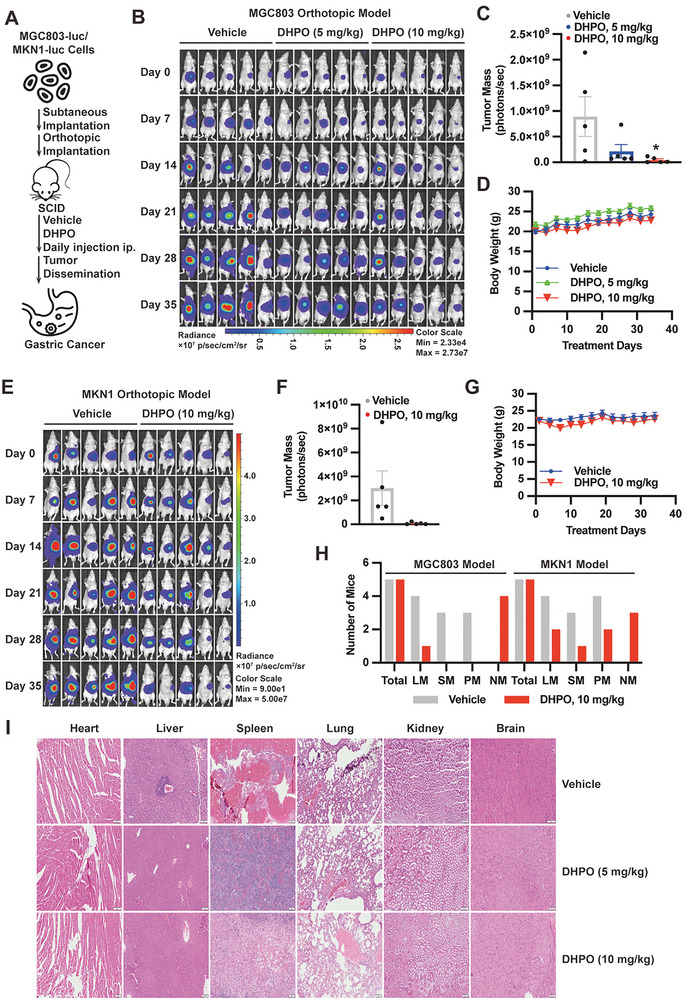
The USP7 inhibitor DHPO demonstrates anti‐cancer efficacy in vivo without causing significant host toxicity. A) A schema depicting the assessment of in vivo efficacy and toxicity of DHPO using orthotopic tumor mouse models derived from MGC803‐Luc and MKN1‐Luc cell lines. B) Bioluminescence imaging demonstrated the efficacy of vehicle and DHPO (5 or 10 mg kg^−1^) in mice bearing MGC803‐Luc orthotopic tumors. C) Quantification of fluorescence intensity was conducted for in vivo imaging of mice treated with vehicle or DHPO (5 and 10 mg kg^−1^). D) Body weight changes monitored in SCID mice receiving vehicle or DHPO treatment. E) Bioluminescence imaging illustrated the differences between mice bearing MKN1‐Luc orthotopic tumors in the vehicle group and those treated with DHPO (10 mg kg^−1^). F) Quantification of fluorescence intensity performed for in vivo imaging of mice in the vehicle and DHPO (10 mg kg^−1^) treatment groups. G) Body weight changes were recorded over time in tumor‐bearing mice treated with vehicle and DHPO (10 mg kg^−1^). H) Statistical analysis presented the numbers of mice having metastasis to liver (LM), spleen (SM), and peritoneum (PM) or no significant metastasis (NM). I) Histological examination of major organs (heart, liver, spleen, lungs, kidneys, and brain) in the vehicle‐ and DHPO (5 or 10 mg kg^−1^)‐treated mice bearing orthotopic tumors.

In vivo efficacy and toxicity of DHPO were evaluated using orthotopic tumor mouse models derived from MGC803‐Luc and MKN1‐luc cell lines (**Figure** **4**A). The experimental groups included vehicle control and DHPO (5 and 10 mg kg^−1^) treatment groups. The tumor growth was significantly reduced in the DHPO‐treated groups compared to the vehicle control (*P* < 0.001) (Figure 4B,C,E,F). Toxicity was evaluated by monitoring changes in body weight. DHPO treatment did not cause significant changes in body weight compared to the vehicle control group at either dose (Figure 4D,G). Metastatic spread is a significant issue in cancers, particularly in GC, where the metastasis commonly occurs in the liver and peritoneum. We investigated the effect of DHPO treatment on metastasis in the liver, peritoneum, and spleen. The results showed that DHPO, at both doses of 5 and 10 mg kg^−1^, effectively suppressed the metastatic tumor nodules in the spleen and liver compared to the vehicle control group (Figure 4H; Figure S2A,B, Supporting Information). Histological analysis of vital organs, including the liver, spleen, and kidney, revealed no signs of toxicity or damage in both DHPO‐treated groups compared to the vehicle control group (Figure 4I).

### 3.4 USP7 Inhibitor DHPO Induced Ferroptosis in GC

**Figure 5:**
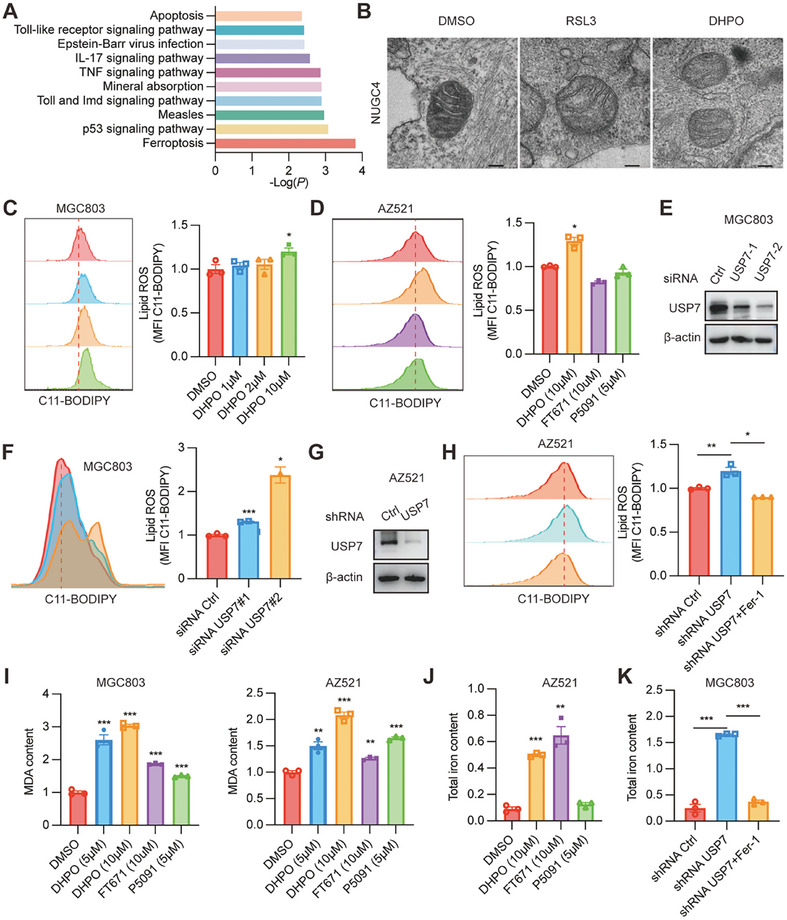
USP7 inhibitor DHPO promotes ferroptosis in gastric cancer. A) TMT proteomic analysis revealed significant dysregulation of ferroptosis in MGC803 cells treated with DHPO compared to DMSO after 24 h. B) Transmission electron microscopy (TEM) images showed alterations in mitochondria morphology in NUGC4 cells upon DHPO treatment. C) Lipid ROS level in MGC803 cells was measured with the treatment of DHPO at different concentrations. D) Lipid ROS level in AZ521 cells was measured with the treatment of DMSO, DHPO, FT671, and P5091. E) Immunoblots for USP7 in MGC803 cells transfected with scrambled or USP7 siRNAs. (F) The representative images (left) and statistical results (right) of lipid ROS after USP7 knockdown in MGC803 cells. G) Immunoblots for USP7 in AZ521 cells transfected with control or USP7 shRNA. H) Lipid ROS measurement after USP7 knockdown with or without treatment of Fer‐1 in AZ521 cells. I) MDA content was determined in MGC803 and AZ521 cells treated with DMSO, DHPO, FT671, and P5091. J) Total iron content was determined in the AZ521 cells treated with DMSO, DHPO, FT671, and P5091. K) Total iron content was determined after USP7 knockdown with or without treatment of Fer‐1 in the MGC803 cells.

To delve deeper into the potential mechanisms of DHPO in GC cells, we conducted TMT‐based proteomics on MGC803 cells treated with DHPO or DMSO for 24 h. A total of 139 proteins displayed significant differential expression (≥1.3‐fold change; *P* <0.05); among them, 104 were upregulated, and 35 were downregulated. The differentially expressed proteins in DHPO‐treated MGC803 cells exhibited primary enrichment in ferroptosis pathways (**Figure** **5**A). Ferroptosis is characterized by mitochondrial membrane disruption, lipid peroxidation, MDA accumulation, and iron overload.^[^ ^23^ ^]^ TEM analysis unveiled shrunken mitochondria with enhanced membrane density in DHPO‐treated NUGC4 cells (Figure 5B). Lipid ROS was increased in MGC803 and AZ521 cells treated with DHPO compared to DMSO control (Figure 5C,D). Interestingly, the previously reported USP7 inhibitors FT671 and P5091 did not impact lipid ROS (Figure 5C,D). To specifically knock down USP7 expression in MGC803 cells, we employed siRNA targeting USP7 (Figure 5E). Remarkably, USP7 knockdown increased lipid ROS generation (Figure 5F). To validate these findings further, we downregulated USP7 expression in AZ521 cells using shRNA targeting USP7 (Figure 5G). Crucially, the lipid ROS level was elevated in USP7 knockdown cells compared to control cells, and this effect was reversible with the ferroptosis inhibitor, Fer‐1 (Figure 5H). The MDA content was observed to accumulate in both MGC803 and AZ521 cells, regardless of whether they were treated with DHPO, FT671, or P5091 (Figure 5I). Iron overload was observed in AZ521 cells treated with DHPO and FT671, but not in cells treated with P5091 (Figure 5J). Iron overload was also observed in MGC803 cells infected with shRNA targeting USP7, which was reversed by Fer‐1 (Figure 5K).

### 3.5 USP7 Regulates Ferroptosis by Deubiquitinating SCD in Response to DHPO Treatment

**Figure 6:**
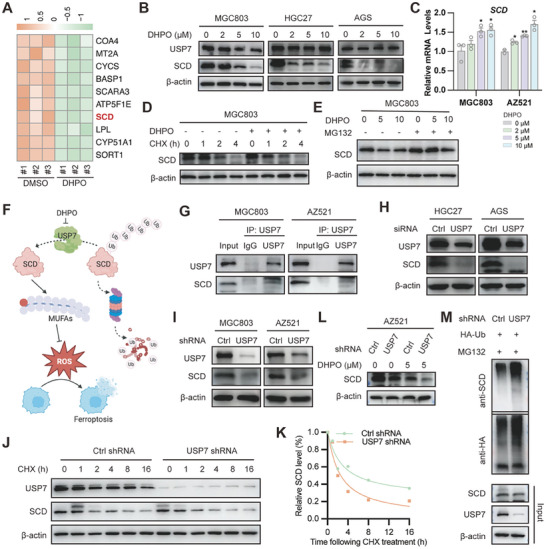
USP7 inhibition induces ferroptosis by targeting the putative substrate SCD. A) Heatmap analysis depicted the differential expression of proteins between DHPO‐ and DMSO‐treated MGC803 cells, with marked downregulation of SCD in the DHPO group. B) Western blot analysis showed the reduced SCD protein levels in MGC803, HGC27, and AGS cell lines treated with escalating concentrations of DHPO for 24 h. C) qRT‐PCR results exhibited a concentration‐dependent increase in SCD mRNA expression in MGC803 and AZ521 cell lines treated with increasing concentrations of DHPO. D) DHPO treatment resulted in an accelerated degradation rate of SCD protein compared to the non‐pretreated group, as assessed by Western blot analysis. E) Treatment of MGC803 cells with MG132 attenuated the degradation of SCD protein by DHPO, as evidenced by Western blot analysis. F) The schematic diagram illustrates that USP7 inhibitor DHPO induces ferroptosis by degrading SCD. G) MGC803 and AZ521 cell lines were harvested for co‐immunoprecipitation assay by using control IgG or USP7 antibody; the immunoprecipitates were then blotted to detect the protein levels of USP7 and SCD. H) The protein level of SCD was detected using Western blot analysis in HGC27 and AGS cell lines transfected with control siRNA or USP7 siRNA. I) The protein level of SCD was detected using Western blot analysis in MGC803 and AZ521 cell lines infected with USP7 shRNA and control shRNA. J,K) USP7 knockdown and control MGC803 cells were treated with 40 µm cycloheximide (CHX) at the indicated time points. The protein degradation rate of SCD was detected using Western blotting and quantified using Image J. L) Knockdown of USP7 using shRNA in AZ521 cells promoted the downregulation of SCD protein expression upon DHPO treatment, as detected by Western blot analysis. M) Control or USP7 knockdown MGC803 cells transfected with HA‐Ub were subjected to co‐immunoprecipitation experiments with HA antibodies, and then Western blotting was used to detect the ubiquitination level of SCD.

To identify the potential substrate of USP7 in the regulation of ferroptosis, we investigated the top 10 proteins downregulated after DHPO treatment, as illustrated in the heatmap (**Figure** **6**A). Among these proteins, SCD drew our attention. As an enzyme involved in monounsaturated fatty acid biosynthesis, SCD plays a pivotal role in regulating lipid peroxidation and ferroptosis.^[^ ^24^, ^25^ ^]^ Inhibition or knockout of SCD increases lipid peroxidation and sensitizes cancer cells to ferroptosis, while overexpression of SCD protects against ferroptosis.^[^ ^26^ ^]^ Existing literature reports that inhibiting SCD decreases cancer cell proliferation and enhances ferroptosis in GC cells.^[^ ^27^ ^]^ This suggests that SCD could be a promising therapeutic target for GC treatment. The impact of the USP7 inhibitor DHPO on SCD expression was assessed using Western blot and qRT‐PCR in MGC803, HGC27, and AGS cells. Following treatment with increasing concentrations of DHPO for 24 h, the SCD protein level was predictably reduced in a concentration‐dependent manner in all cell lines (Figure 6B), while the mRNA expression of SCD was increased in a concentration‐dependent manner in MGC803 and AZ521 cells (Figure 6C). Furthermore, DHPO shortened the half‐life of SCD protein when new protein synthesis was inhibited by cycloheximide (Figure 6D). The degradation of SCD protein was hindered by MG132 (Figure 6E), confirming that USP7 sustains SCD protein through the ubiquitination‐proteasome pathway. Based on these findings, we propose that USP7 regulates ferroptosis by deubiquitinating SCD (Figure 6F). Co‐IP validated the protein interaction between USP7 and SCD (Figure 6G). The expression level of SCD was decreased when USP7 was knocked down using siRNA or shRNA targeting USP7 (Figure 6H,I). Compared to the control group, knocking down USP7 in MGC803 cells reduced the half‐life of the SCD protein from 5 h to 2.5 h (Figure 6J,K), indicating USP7's involvement in SCD protein stabilization. Knocking down USP7 resulted in an increase in the inhibitory effect of DHPO on SCD in AZ521 cells (Figure 6L), further confirming USP7 as the target of action for DHPO. Furthermore, USP7 knockdown increased the polyubiquitin chain conjugated to SCD, suggesting that USP7 regulates SCD deubiquitination (Figure 6M). Collectively, our data suggest that USP7 inhibition by DHPO increases SCD ubiquitination and accelerates its proteasomal degradation.

### 3.6 USP7 Promoted Tumorigenesis by Increasing SCD Level and Served as an Independent Prognostic Biomarker

**Figure 7:**
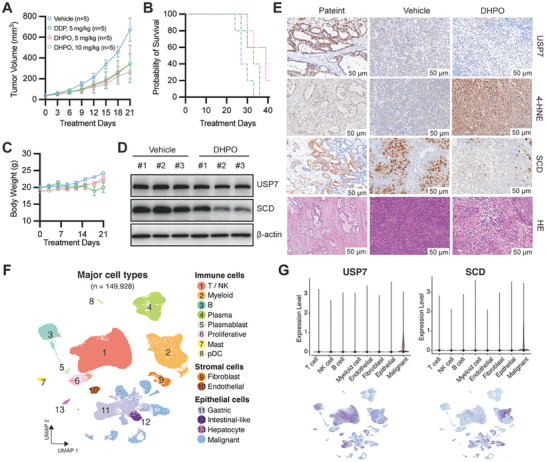
USP7‐SCD axis is negatively correlated with overall survival of PDX‐bearing mice. (A‐C) Tumor volume A), survival time B), and body weight C) were evaluated in mice bearing patient‐derived xenografts (PDX) treated with vehicle, DHPO (5 and 10 mg kg^−1^), or cisplatin (5 mg kg^−1^). D) SCD protein expression was assessed by Western blot analysis in PDXs from control and DHPO‐treated groups. E) Immunohistochemistry (IHC) images demonstrated USP7, 4‐HNE, and SCD expression, and Hematoxylin and Eosin staining (HE) in PDX from the control and DHPO‐treated groups. F) scRNA‐seq analyses showed that USP7 and SCD were highly expressed in gastric cancer cells.

Consistent with the results obtained from the orthotopic tumor mouse models, DHPO treatment at both doses in the GC PDX mouse model resulted in a significant decrease in tumor weight compared to the control group (**Figure** **7**A). We also studied the impact of DHPO on overall survival. The results showed that treatment with the DHPO significantly prolonged survival compared to the control and cisplatin‐treated groups (Figure 7B). On Day 36, all mice in the control group had died, while more than half of the mice in the USP7 inhibitor‐treated group were alive (Figure 7B). The cisplatin‐treated group showed a similar survival rate to the control group (Figure 7B), indicating that the traditional chemotherapeutic agent had limited effects on extending overall survival in this model. Further analysis showed that the USP7 inhibitor‐treated groups had no significant adverse effects on body weight compared to the control group (P <0.05) (Figure 7C). In contrast, the cisplatin‐treated group showed a significant reduction in body weight compared to all other groups (P <0.05) (Figure 7C), reflecting the well‐known cytotoxic effects of cisplatin. Overall, our results suggest that DHPO treatment at both doses of 5 and 10 mg kg^−1^ effectively inhibits tumor growth without causing significant toxicity or damage to vital organs. 4‐HNE, a highly toxic and most abundant stable end product of lipid peroxidation, has been implicated in ferroptosis.^[^ ^28^ ^]^ IHC results indicated that, compared to the control group, the expression of 4‐HNE protein in tumor tissues of mice treated with DHPO increased (Figure 7E), confirming the in vivo induction of ferroptosis in GC by DHPO. Consistent with the findings in GC cells in vitro, we observed a significant downregulation of SCD expression in the GC PDX tumors by Western blot (Figure 7D) and IHC (Figure 7E) upon USP7 inhibition. This finding suggests that the observed downregulation of SCD by USP7 inhibition may contribute to the suppression of tumor growth in vivo. To further investigate the oncogenic role of USP7 and SCD spatially and temporally, we utilized a scRNA‐seq cohort^[^ ^29^ ^]^ and identified the GC cell populations. Surprisingly, we found that both USP7 and SCD exhibited high expression in malignant cells (Figure 7F), further supporting the notion that USP7 and SCD are potent oncogenes in GC. Therefore, DHPO appears to be a potentially safer alternative or complementary treatment option for GC. This information is critical in the development of effective cancer therapies that can extend survival and improve the quality of life of cancer patients.

## 4 Discussion

Ferroptosis, an iron‐dependent form of cell death characterized by lipid peroxide accumulation and redox imbalance, holds promise in cancer therapeutics, particularly in GC. Prior research has unveiled the intricate involvement of ferroptosis in GC, linking it to cell proliferation, metastasis, and chemotherapy resistance. Key genes and non‐coding RNAs, such as BAP31,^[^ ^30^ ^]^ GPX4,^[^ ^31^ ^]^ STAT3,^[^ ^32^ ^]^ ATF2,^[^ ^33^ ^]^ and ARF6,^[^ ^34^ ^]^ play pivotal roles in orchestrating ferroptosis in GC, shedding light on its significant contribution to GC development and providing novel avenues for therapeutic exploration.

Several studies are underway to identify potential GC treatment candidates. Tanshinone IIA, for instance, inhibits GC cell growth and metastasis by modulating lipid peroxide and glutathione levels.^[^ ^35^ ^]^ *Actinidia chinensis* Planch demonstrates anti‐proliferative and anti‐migratory effects on GC cells, concurrently downregulating GPX4 expression.^[^ ^36^ ^]^ Polyphyllin B demonstrated the ability to induce ferroptosis in GC cells by modulating GPX4, TFR1, NOCA4, and FTH1 expression.^[^ ^37^ ^]^ Amentoflavone, a multifunctional biflavonoid, suppresses proliferation and induces ferroptotic cell death in GC cells via the miR‐496/ATF2 axis.^[^ ^38^ ^]^ Despite notable progress, there remain unknown aspects of the specific mechanisms of ferroptosis in GC, warranting further research. Additionally, the development of more effective treatment strategies leveraging this knowledge represents a crucial direction for future studies.

In recent years, USP7 has emerged as a pivotal regulator of ferroptosis, with studies shedding light on its role in various contexts. Tang et al. delved into its participation in cardiac ischemia/reperfusion (I/R), revealing a correlation between elevated USP7, p53, and TfR1 levels and increased ferroptosis. Inhibiting USP7 mitigated myocardial I/R injury.^[^ ^39^ ^]^ Dong et al. explored renal I/R injury, demonstrating that USP7 inhibition attenuated I/R‐induced renal injury by inhibiting ferroptosis through decreasing ubiquitination of TBK1 and promoting DNMT1‐mediated methylation of FMR1.^[^ ^40^ ^]^ A study on spinal cord injury associated downregulated USP7 and upregulated HMOX‐1 with improved motor function recovery and ferroptosis prevention.^[^ ^41^ ^]^ These findings collectively emphasize the critical regulatory influence of USP7 in ferroptosis across various pathological conditions, presenting potential therapeutic avenues. However, the specific role of USP7 in regulating ferroptosis in GC requires further exploration.

In our study, we identified DHPO as an allosteric site covalent inhibitor of USP7. Using this inhibitor, we discovered that DHPO suppresses GC growth and metastasis by downregulating SCD and inducing ferroptosis. Through siRNA and shRNA targeting USP7, we demonstrated that USP7 inhibition promotes ferroptosis by deubiquitinating SCD. Interestingly, previously reported USP7 inhibitors, such as FT671 and P5091, exhibited differential effects on ferroptosis‐related indicators, including the accumulation of lipid ROS, MDA content, and iron overload. These variations may be attributed to the distinct binding sites and inhibitory mechanisms among these three different USP7 inhibitors. Therefore, co‐crystallization studies are warranted to achieve a deeper understanding of the DHPO‐USP7 interaction. Moreover, guided structural modifications hold promise for developing more effective molecules with improved biological activity and reduced off‐target effects for clinical translation. Prior to clinical application, higher‐level evidence verification in vivo, is essential for elucidating the relationship between USP7, SCD, and ferroptosis.

In conclusion, this study provides insights into USP7 as a therapeutic target in GC by inhibiting ferroptosis. The findings pave the way for potential interventions in GC therapy, leveraging USP7 degradation and ferroptosis induction.

## Conflict of Interest

The authors declare no conflict of interest.

## Author Contributions

X.G. and Y.W. contributed equally to this work. J.J.Q., W.Z., and XD.C. designed the experiments and supervised the project; X.G. and Y.W. performed most of the experiments with the assistance from W.Y., Y.W., Y.L., B.Z., C.H., and L.Y.; X.G., E.D., X.D. and X.L. performed data analysis; X.G. and J.J.Q. wrote and edited the manuscript. K.M. and B.C. made significant revisions. All authors read and approved the final paper.

## Supporting information

Supporting Information

## Data Availability

The data that support the findings of this study are available from the corresponding author upon reasonable request.
