# Supplementary material for: Blocking Ubiquitin‐Specific Protease 7 Induces Ferroptosis in Gastric Cancer via Targeting Stearoyl‐CoA Desaturase
Source: Adv Sci (Weinh). 2024 Mar 9;11(18):2307899. doi: 10.1002/advs.202307899 (PMC11095140; doi:10.1002/advs.202307899)
Supplement: Supplementary file 1 — Supporting Information [file ADVS-11-2307899-s001.pdf]

ADVANCED SCIENCE
Open Access

Supporting Information

for *Adv. Sci.*, DOI 10.1002/advs.202307899

Blocking Ubiquitin-Specific Protease 7 Induces Ferroptosis in Gastric Cancer via Targeting Stearoyl-CoA Desaturase

Xiaoqing Guan, Yichao Wang, Wenkai Yu, Yong Wei, Yang Lu, Enyu Dai, Xiaowu Dong, Bing Zhao, Can Hu, Li Yuan, Xin Luan, Kai Miao, Bonan Chen, Xiang-Dong Cheng, Weidong Zhang* and Jiang-Jiang Qin**

Supplementary figures

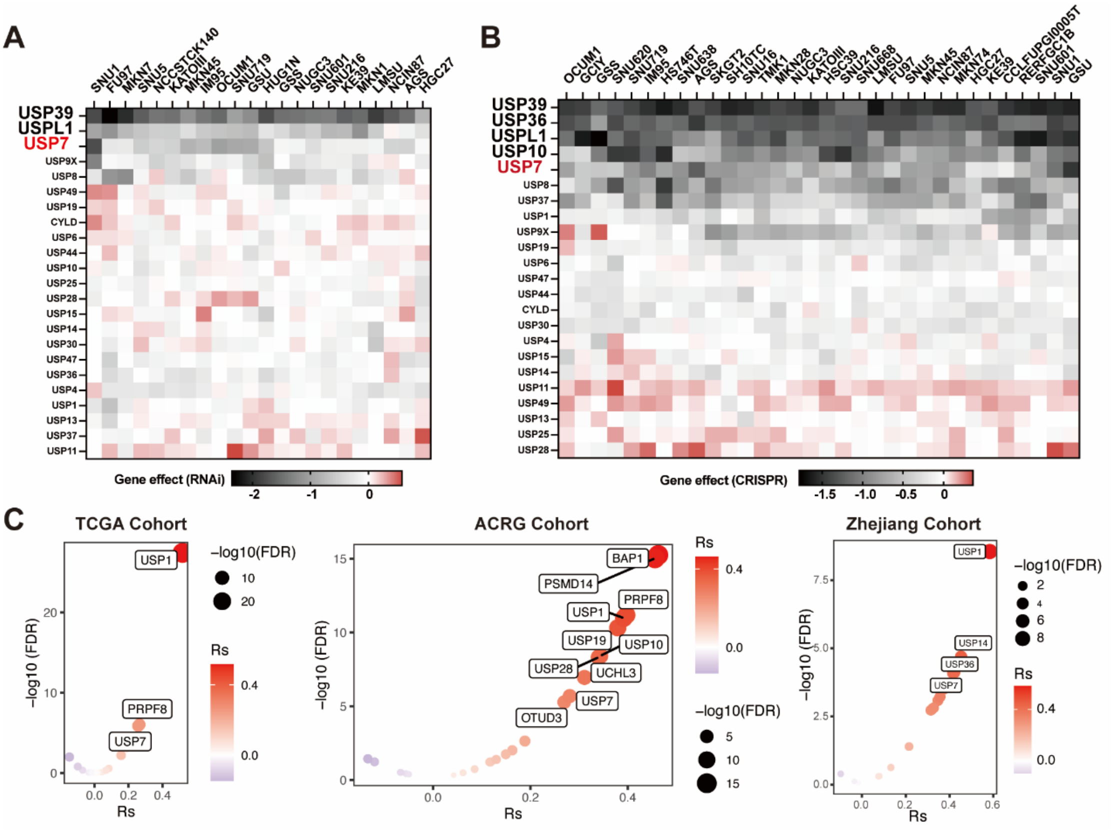

Figure S1 Deubiquitinating enzymes play important roles in gastric cancer. (**A**) and (**B**) Heatmap showing the deubiquitinating enzymes essentiality in gastric cancer cell lines from publicly available RNAi screening (**A**) and CRISPR-Cas9 screening (**B**). Essential genes are shown in gray, and nonessential genes are depicted in red. (**C**) Pearson correlation coefficient between Deubiquitinating enzymes and *Ki-67* mRNA expression in TCGA, ACRG, and Zhejiang cohorts.

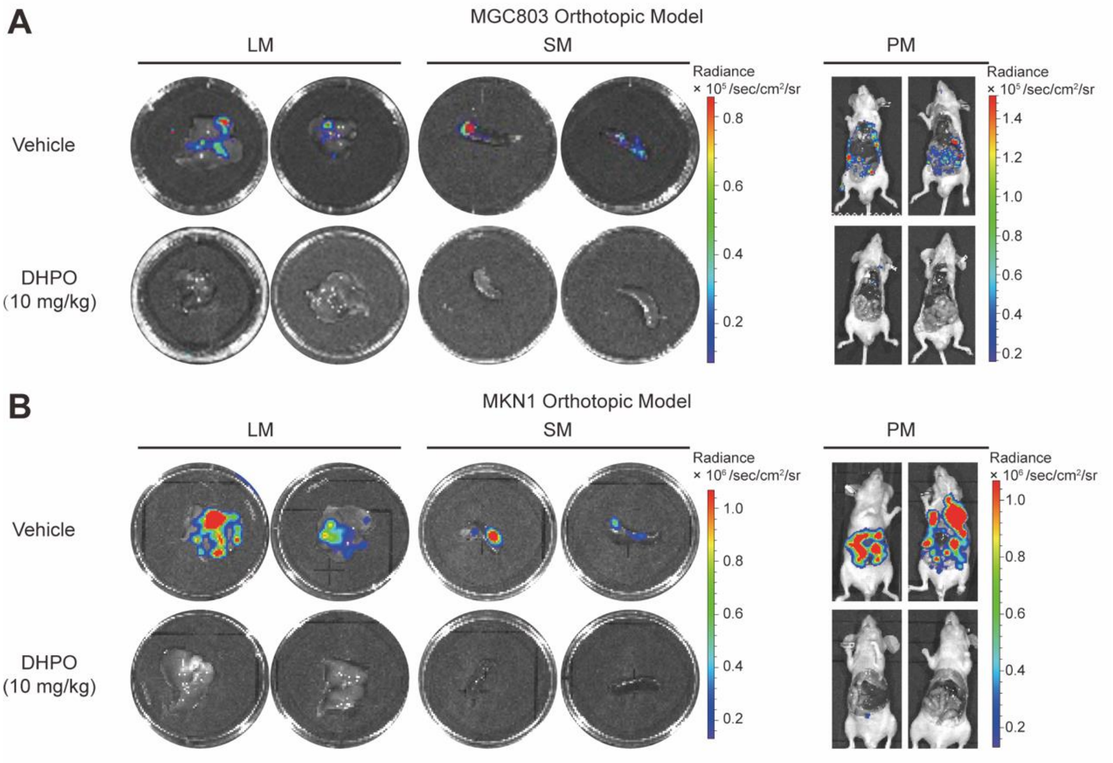

Figure S2 DHPO suppresses metastasis in orthotopic tumor mouse models. (**A and B**) Visualization of liver, spleen, and peritoneal metastases in orthotopic tumor SCID mouse models derived from MGC803 and MKN1 cells treated with vehicle control or DHPO (10 mg/kg).
